# Magnitude and determinants of inappropriate prescribing of antibiotics in dentistry: a nation-wide study

**DOI:** 10.1186/s13756-023-01225-z

**Published:** 2023-03-20

**Authors:** Almudena Rodríguez-Fernández, Olalla Vázquez-Cancela, María Piñeiro-Lamas, María Teresa Herdeiro, Adolfo Figueiras, Maruxa Zapata-Cachafeiro

**Affiliations:** 1 Department of Preventive Medicine and Public Health, University of Santiago de Compostela, 15706 Santiago de Compostela, A Coruña, Spain; 2 Department of Preventive Medicine, Santiago de Compostela University Teaching Hospital, Santiago de Compostela, Spain; 3 Consortium for Biomedical Research in Epidemiology and Public Health (CIBER en Epidemiología y Salud Pública-CIBERESP), Santiago de Compostela, Spain; 4 Institute of Health Research of Santiago de Compostela, Santiago de Compostela, Spain; 5 Department of Medical Sciences, Institute of Biomedicine - iBiMED, University of Aveiro, Aveiro, Portugal

**Keywords:** Antibiotic, Dentist, Prescribing, Knowledge, Attitudes

## Abstract

**Background:**

Dentist play an important role in misuse of antibiotics. Identification of the dental activities linked to the misuse of antibiotics is important for improving dentists’ prescribing quality. The aim of the study was to quantify the magnitude of inappropriate antibiotic prescribing by dentists in Spain and identify the characteristics, knowledge and attitudes that influence prescribing quality.

**Material and methods:**

We conducted a cross-sectional, questionnaire-based study on dentists in Spain, assessing prescribing quality (dependent variable) on the basis of their responses about the prescription of antibiotics in 14 clinical situations. As the independent variables, we assessed professional characteristics and attitudes (lack of knowledge, fear, complacency, scheduling problems, and economic benefit) measured on a Likert scale. Odds Ratios (OR) (95%CI) were calculated using logistic regression.

**Results:**

A total of 878 participants were included in the analysis. Half of all dentists displayed inappropriate antibiotic prescribing habits in more than 28.6% (10/14) of the clinical situations posed (interquartile range 57–79%). Prescribing quality increased when resistance was perceived as a public health problem (OR 0.88, 95% CI: 0.79–0.97), and decreased in response to fear (OR 1.12, 95% CI:1.07–1.18) or the pursuit of economic benefit (OR 1.07, 95% CI 1.01–1.14). Having over 30 years’ experience (OR 4.58, 95% CI:1.80–12.48) and/or practising in the field of prosthodontics as opposed to endodontics (OR 2.65, 95% CI:1.26–5.71) were associated with worse prescribing quality.

**Conclusions:**

Antibiotics are the most commonly prescribed drugs in dentistry, and in many cases this prescription is inappropriate. Our findings shows that modifiable factors influence prescribing quality among dentists in Spain. These may be use for designing educational and training programmes for dentists.

**Supplementary Information:**

The online version contains supplementary material available at 10.1186/s13756-023-01225-z.

## Introduction

Bacterial resistance is a major public health problem, in that it increases mortality, morbidity and healthcare costs [1]. It has been estimated that it could account for as much as $3 trillion of lost gross domestic product by 2050 [2]. Indeed, the WHO considers it to be one of the greatest threats to public health worldwide [3]. Despite the fact that the development of such resistance is a natural and inevitable phenomenon, it is further exacerbated by excessive and inappropriate use of antibiotics. A number of agents are involved in such misuse, ranging from health professionals, physicians and dentists to the general population [4–7].

In dentistry, antibiotics are the most commonly prescribed drugs [8] although they are only indicated for the treatment of processes in which the patient’s immune defences are unable to control the infection, or where there is evidence of systemic involvement [7, 9, 10]. Even so, dentists prescribe around 10% of total antibiotics consumed [11, 12], and it is estimated that over 70% of these prescriptions would not be considered appropriate [13, 14]. It therefore follows that identifying which factors are associated with inappropriate prescribing would enable purpose-designed educational interventions to be implemented [15], and antibiotic use optimised in this group of professionals. Yet, we have been unable to locate studies that quantify the influence of knowledge and/or attitudes on prescribing quality in dentistry.

The aims of this study were therefore: (i) to quantify the magnitude of inappropriate antibiotic prescribing by dentists in Spain; (ii) to ascertain their socio-demographic characteristics; and (iii) to identify the knowledge and attitudes that influence antibiotic-prescribing quality in dentistry.

## Methods

### Study setting

The study was undertaken in Spain, where dental care is provided by the country’s National Health System (NHS) or private dental clinics. As the NHS solely provides oral surgery and pharmacological treatment of acute processes, all remaining treatments are offered by private clinics. As a result, these deliver dental care to 85.5% of the population [16] and employ over 90% of all registered dentists in Spain [17]. The profession of dentists is regulated and in order to practice, it is necessary to have a degree in dentistry or to be a medical specialist in stomatology.

### Study design and population

We conducted a cross-sectional, questionnaire-based study in accordance with the STROBE (Strengthening the Reporting of Observational Studies in Epidemiology) guidelines [18], covering all registered dentists practising in Spain. As it is a cross-sectional study, it impossible to differentiate between cause and effect. However, knowledge and attitudes are fairly stable variables over time. Therefore, a cross-sectional measure can be considered equivalent to a longitudinal measure*.* Potential population was formed by the 38.809 dentists registered and working in Spain, according to 2019 data. By way of inclusion criterion, study subjects were required to be: (i) graduates in dentistry or oral medicine specialists (stomatologists); (ii) registered with one of the dental professional associations (*colegio profesional de dentistas*) in Spain; and (iii) gainfully employed at the time of completing the questionnaire. Given that the designated objective of the study was to assess prescribing quality, monthly prescription of fewer than 5 antibiotics was established as the exclusion criterion [19, 20].

According to studies conducted on this population using the same data-collection method [21, 22], initial estimates indicated an expected participation of 1–2% of the total number of dentists, 38,809, yielding a sample size of 380–760. Assuming a prevalence of inappropriate prescribing of 70% [11] a sample of 800 subjects would ensure a precision of over 3.5%. Indeed, previous studies by our group using similar scales and variables, and a sample size of 286 [23], managed to detect statistically significant differences between attitudes and inappropriate dispensing of antibiotics, with a very similar prevalence of inappropriate dispensing (65%).

### Measures

We used an anonymous, self-administered online questionnaire (available as Additional file 1: Table S1), made up of 26 questions in 4 blocks:

The first block assessed participants’ degree of agreement, measured on a Likert scale scored from 0 to 10, with seven items addressing attitudes to antibiotics and bacterial resistance, linked to *lack of knowledge, responsibility of others, fear, complacency, scheduling problems/time, economic benefit/lack of patient trust*;

- The second block assessed antibiotic prescribing habits in different scenarios;
- The third block gathered personal and professional data; and,
- The last block consisted of open-ended questions, in which the participants could make suggestions for improvement, or comment on difficulties encountered while completing the questionnaire.

Reading and completing the questionnaire took approximately 10 min and only required a device with an Internet connection.

### Questionnaire design, validity, and pilot study

The questionnaire was designed on the basis of previous studies, using fully-validated questionnaires, expert opinion, and the indications of the Aljarafe Antimicrobial Treatment Guidelines (*Guía Terapéutica Antimicrobiana Aljarafe*), the reference guide in Spain for primary care antibiotic optimisation programmes at the date of data-collection [24–29].

A multidisciplinary team, separate from and independent of the research team, (consisting of dentists, pharmacists, medical specialists in and microbiology, specialist in public health, and psychologists), conducted an analysis of logical and content validity. Questions that were not self-explanatory were changed or deleted, and others judged to be of possible interest in light of the study objectives, were added.

A pilot study was conducted with the first 50 responders, with the aim of assessing the need to change one or more questions. A comments section was included to record any possible drawbacks or difficulties experienced, and in that way allow for amendments to the final questionnaire.

### Procedure

The questionnaire was disseminated from June to September 2021 via social networks (Twitter, Whatsapp and Telegram), web sites of dental professional associations, scientific dental societies, and the National Antibiotic Resistance Plan [30]. The link was accompanied by a letter of presentation which described the overall aim of the study, outlined the instructions for completing the questionnaire, and asked professionals to circulate it among their colleagues.

### Definition of variables

The main dependent variable was defined as overall prescribing quality, created on the basis of 14 clinical situations, in which the responder was required to state whether he/she would prescribe antibiotics (second block). Suitability of antibiotic prescribing was classified as appropriate/inappropriate in line with the Aljarafe Guidelines [29], a nationwide reference guide at the date of study. All situations were intended for a non-immunocompromised patient.

The secondary dependent variables were prescribing quality in: (i) pulp diseases; (ii) emergency visits; and (iii) preventive treatment of infections. Table 1 shows which clinical situations were grouped into each of these categories.

**Table 1:** Antibiotic prescribing quality among dentists

Pulp diseases	Antibiotic needed?*	Correct n (%)	Incorrect n (%)
Symptomatic irreversible pulpitis (moderate/severe preoperative symptoms)	No	683 (77.79)	195 (22.21)
Symptomatic irreversible pulpitis with acute periapical periodontitis (moderate/ severe preoperative symptoms)	No	403 (45.90)	475 (54.10)
Necrotic pulp with asymptomatic apical periodontitis (no swelling, no/mild preoperative symptoms)	No	735 (83.71)	143 (16.29)
Necrotic pulp with acute apical periodontitis (no swelling, moderate/severe preoperative symptoms)	No	415 (47.27)	463 (52.73)
Necrotic pulp with chronic apical periodontitis (sinus trac present, no swelling, no/mild preoperative symptoms)	No	612 (69.70)	266 (30.30)
Necrotic pulp with acute apical abscess (swelling present, moderate/ severe preoperative symptoms)	Yes	819 (93.28)	59 (6.72)
Overall (all correct/all incorrect)		181 (20.62)	0
*Dental emergencies*			
Postoperative pain after instumentation or obturation	No	778 (88.61)	100 (11.39)
Avulsion	Yes	521 (59.34)	357 (40.66)
Incision and drainage localized intraoral swelling	No	436 (49.66)	442 (50.34)
Postoperative pain	No	805 (91.69)	73 (8.31)
Pericoronitis (no swelling or systemic symptoms)	No	578 (65.83)	300 (34.17)
Necrotising ulcerative gingivitis	No	247 (28.13)	631 (81.87)
Overall (all correct/all incorrect)		32 (3.64)	4 (0.46)
*Prevention of infections*			
Prevent infection after dental extraction	No	692 (78.82)	186 (21.18)
Antibiotic prophylaxis to prevent dental implant failure (routine situations)	No	480 (54.67)	398 (45.33)
Overall (all correct/all incorrect)		406 (46.24)	112 (12.76)

*The correct response was classified as correct or incorrect in accordance with the Aljarafe Guidelines, a nationwide reference guide at the date of study [28]

All the dependent variables were categorised into dichotomous variables (appropriate prescribing = 1 versus inappropriate prescribing = 0), with the cut-off point set as the median of correct responses in each of the variables made up of more than 2 items. For preventive prescribing (2 items), a good prescriber was defined as one who responded correctly to both items.

As independent variables, we considered participants’ personal and professional characteristics, as well as their degree of agreement with items addressing knowledge and attitudes.

### Statistical analysis

We performed a descriptive analysis (n, %) of questions relating to personal and professional data, and calculated the 25th, 50th and 75th percentiles of the degree of agreement with attitudes. The influence of the independent variables on the main and secondary dependent variables was studied using logistic regression. The influence of independent variables on primary and secondary dependent variables was studied by logistic regression. This analysis allows to provide a magnitude of effect (expressed in odds ratio) and adjusted for other potential confounding variables.

We constructed the following 2 blocks of models: (i) personal and professional variables; and (ii) attitudes. For the first block, we adjusted for all the other independent variables included in the model. In the analysis of attitudes, we adjusted for potentially confounding personal and professional characteristics (those with a *p-*value < 0.2, which led to a change of more than 10% in the coefficient). Results were expressed as odds ratios with their 95% confidence intervals.

## Results

### Participation and characteristics of the study population

**Fig. 1:**
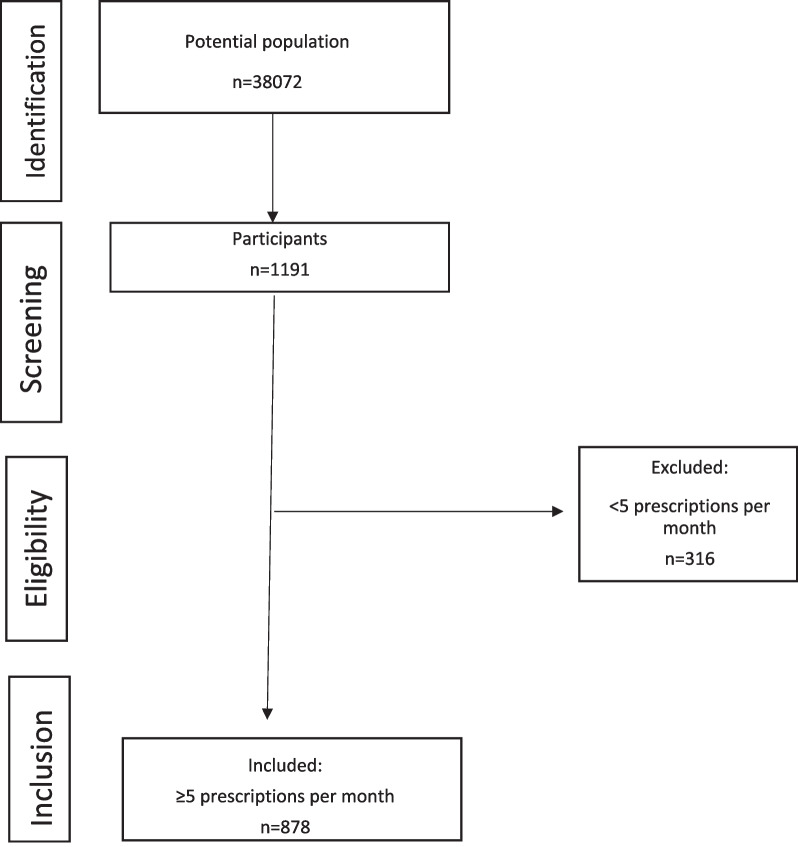
Flow chart

After the pilot study, no amendments had to be made to the questionnaire. In all, 1191 dentists answered the questionnaire (Fig. 1), and of these 316 were excluded for prescribing 5 or fewer antibiotics per month. Finally, 878 dentists were considered for analysis purposes.

Participants’ personal and professional characteristics are shown in Table 2: 58.2% were women; 46.3% had been practising for 21 years or more as a dentist; and 72.1% carried out their professional activity at a privately owned clinic.

**Table 2:** Influence of personal and professional characteristics on prescribing quality

	Total	Prescribing quality*		OR (95% IC)**
		Appropiate n (%)	Inappropiate n (%)	
*Sex*				
Male	358 (41.8)	177 (49.4)	181 (50.6)	1
Female	498 (58.2)	259 (52.0)	239 (48.0)	1.19 (0.82–1.72)
*Academic background*				
Degree in Denistry	690 (79.1)	360 (52.2)	330 (47.8)	1
Stomatologist	117 (13.4)	59 (50.4)	58 (49.6)	0.55 (0.26–1.15)
Medical Doctor and Graduate in Denistry	65 (7.5)	29 (44.6)	36 (55.4)	0.84 (0.39–1.78)
*How many years have you been practising as a dentist?*				
< 5	84 (9.6)	53 (63.1)	31 (36.9)	1
5–10	133 (15.2)	72 (54.1)	61 (45.9)	2.07 (0.96–4.76)
11–20	252 (28.9)	128 (50.8)	124 (49.2)	2.38 (1.16–5.15)
21–30	249 (28.5)	126 (50.6)	123 (49.4)	2.61 (1.25–5.74)
> 30	155 (17.8)	69 (44.5)	86 (55.5)	4.58 (1.80–12.48)
*In what type of clinic do you work?*				
Primary health centre	77 (8.8)	48 (62.3)	29 (37.7)	1
Private clinic	633 (72.1)	315 (49.8)	318 (50.2)	2.35 (1.14–5.00)
University	8 (0.9)	1 (12.5)	7 (87.5)	–
Primary health centre and private clinic	83 (9.5)	41 (49.4)	42 (50.6)	1.87 (0.81–4.38)
Primary health centre and university	8 (0.9)	6 (75.0)	2 (25.0)	0.86 (0.11–4.64)
Private clinic and university	52 (5.9)	30 (57.7)	22 (42.3)	1.87 (0.74–4.81)
All	5 (0.6)	3 (60.0)	2 (40.0)	1.33 (0.06–16.01)
Others	12 (1.4)	6 (50.0)	6 (50.0)	1.94 (0.38–9.30)
*Have you completed any of the following postgraduate studies?*				
Master’s degree in endodontics	90 (14.7)	54 (60)	36 (40.0)	1
Master’s degree in periodontics	67 (10.9)	38 (56.7)	29 (43.3)	1.11 (0.56–2.21)
Master’s degree in surgery	222 (36.2)	112 (50.5)	110 (49.6)	1.49 (0.88–2.56)
Master’s degree in pediatric dentistry	31 (5.0)	22 (71.0)	9 (29.0)	0.66 (0.25–1.62)
Master’s degree in prosthodontics	50 (8.1)	18 (36.0)	32 (64.0)	2.65 (1.26–5.71)
Master’s degree in public health	46 (7.5)	28 (60.9)	18 (39.1)	1.37 (0.57–3.30)
Other	108 (17.6)	57 (52.8)	51 (47.2)	1.30 (0.71–2.39)

*Main dependent variable, which covers 14 clinical situations. To consider prescribing appropriate, the cut-off point was set as equal to or higher than the median of correct responses (≥ 10 correct responses out of 14 questions)

**Adjusted for all the other variables included in the table

### Prescribing quality

For the variable of overall prescribing quality (principal variable), the median of correct responses was 10 (interquartile range (IR) 8–11) out of the 14 situations posed, indicating that half of all dentists displayed inappropriate antibiotic prescribing habits in more than 28.6% (10/14) of these clinical situations (IR 57–79%). For the variables of pulp therapy and emergency prescriptions the median was 4 out of a total of 6 situations (IR 3–5 and IR 3–5 respectively). For the variable of preventive prescribing, 46.24% of participants were classified as good prescribers.

Table 1 shows the proposed clinical situations, categorised by the disease to which they refer, i.e., pulp, emergency, and prevention of infections. It also shows the percentage of correct and incorrect responses obtained. It also shows the percentage of correct and incorrect responses obtained. Highlights that 54.10% (95% CI: 50.79–57.39) of responders would prescribe antibiotics for acute apical periodontitis, 8.31% (95% CI: 6.66–10.33) for postoperative pain, and 45.33% (95% CI: 42.07–48.64) would prescribe them for the prevention of dental implant failure.

### Influence of socio-demographic and professional factors on antibiotic prescribing

Table 2 shows the influence of personal and professional characteristics in terms of their association with overall prescribing quality (main variable). Years of experience, working in a private clinic, and having taken a masters degree in prosthodontics, showed worse results in prescribing quality. The fact of having practised for more than 30 years increased the risk of inappropriate prescribing (OR 4.58; 95% CI: 1.80–12.48) 4.5 fold as compared to having practised for less than 5 years. No association was found between sex and prescribing quality, or with academic qualifications.

### Influence of attitudes and knowledge on prescribing quality

#### Overall prescribing quality

Table 3 shows participants’ degree of agreement as regards knowledge and attitudes and their influence on prescribing quality. In general, dentists showed a high degree of agreement about the attitudes evaluated. The greatest discrepancy was observed in the item that assessed fear (IR = 5).

**Table 3:** Analysis adjusted for the influence exerted by attitudes to and opinions about prescribing-on-prescribing quality

Attitudes	Appropiate prescription of antibiotics by dentist***										
	Percentiles			Overall		In pulp diseases**		In emergency medical visitis**		In prevention of infection*	
	25	50	75	OR (CI95%)	*p*- value	OR (CI95%)	*p*- value	OR (CI95%)	*p*- value	OR (CI95%)	*p*- value
Antibiotic resistance is an important public health problem. *(Knowledge)*	9	10	10	0.88 (0.79–0.97)	0.0141	0.86 (0.77–0.96)	0.0091	0.95 (0.84–1.07)	0.4226	0.89 (0.79–0.99)	0,0358
Dentists play an important role in the rational use of antibiotics. *(External responsibility)*	8	10	10	0.92 (0.83–1.03)	0.1368	1.03 (0.92–1.14)	0.6307	0.89 (0.78–1.01)	0.0771	0.93 (0.83–1.04)	0,1847
The prescribing of an antibiotic to a patient does not influence the development of resistance. (*Knowledge*)	0	1	3	1.02 (0.98–1.07)	0.3062	1.04 (0.99–1.09)	0.0876	0.98 (0.93–1.03)	0.4864	1.02 (0.97–1.07)	0,5094
When I doubt whether a patient has a bacterial infection, I prefer to prescribe an antibiotic. *(Fear)*	2	5	7	1.12 (1.07–1.18)	< 0.0001	1.09 (1.04–1.14)	0.0006	1.09 (1.04–1.15)	0.0007	1.11 (1.05–1.17)	0,0001
I often prescribe antibiotics because patients demand them from me. *(Complacency)*	0	1	3	1.02 (0.96–1.08)	0.4691	0.98 (0.93–1.04)	0.5069	1.07 (1.00–1.15)	0.0542	1.05 (0.99–1.12)	0,1230
I sometimes prescribe antibiotics knowing that they are not indicated, due to lack of time during the visit. *(Schedule/time)*	0	0	2	1.04 (0.98–1.11)	0.1789	1.00 (0.94–1.06)	0.9573	1.03 (0.96–1.10)	0.4009	1.01 (0.95–1.07)	0,8100
I tend to prescribe antibiotics so that my patients trust that I am doing everything possible. *(Economic benefit)*	0	0	2	1.07 (1.01–1.14)	0.0203	1.03 (0.97–1.09)	0.3084	1.10 (1.03–1.18)	0.0083	1.05 (0.99–1.12)	0,1384

*Adjusted for potentially confounding variables (sex, work setting and years of experience)

**Adjusted for potentially confounding variables (sex and years of experience)

***Appropriate prescribing cut-off point: the median of correct responses in each of the variables made up of more than 2 items. Overall: ≥ 10 correct responses in 14 questions; pulp diseases and emergency disorders ≥ 4 correct responses in 6 questions; preventive prescription (2 items), a good prescriber was defined as one who responded correctly to both items

Prescribing quality increased significantly among those who consider resistance to be an important public health problem (knowledge) (OR 0.88, 95% CI: 0.79–0.97, *p* = 0.01). The pursuit of economic benefit increased the risk of inappropriate prescribing by 7% (OR 1.07, 95% CI: 1.01–1.14, *p* = 0.02) for every one-unit increase on the Likert scale. Similarly, a one-unit increase on the Likert scale in the item that assessed fear led to a 1.12-fold decrease in the probability of appropriate prescribing (OR 1.12; IC95% 1.07–1.18, *p* < 0.001).

There was no significant effect on the items that evaluated external responsibility, complacency or lack of time.

#### Prescribing quality by clinical indication

In pulp diseases (Table 3), fear was observed to worsen prescribing quality significantly, with a one-unit increase on the Likert scale leading to a 1.09-fold increase (OR 1.09, 95% CI: 1.04–1.14, *p* < 0.01) in the probability of inappropriate prescribing in these types of clinical situations.

Insofar as dental emergencies were concerned, economic benefit increased the risk of inappropriate prescribing by 10% (OR 1.10, 95% CI: 1.03–1.18, *p* = 0.008) for every one-unit increase on the Likert scale. Complacency towards the patient was also identified with inappropriate prescribing in dental emergencies, though the results were not significant (*p* = 0.0542).

In the case of situations of prevention of infections, fear was the principal factor that influenced inappropriate prescribing, with every one-unit increase on the Likert scale showing an increase of 1.11 (OR 1.11, 95% CI: 1.05–1.17, *p* < 0.001) in the probability of an inappropriate prescription being issued.

## Discussion

To our knowledge, this is the first paper to show that knowledge and attitudes influence prescribing quality among dentists. Our results indicate that, while modifiable factors, such as knowledge, raise prescribing quality, when factors such as fear or the pursuit of economic benefit increase, prescribing quality then decreases. Other factors, such as experience, work setting or having received rehabilitation-centred training, have likewise been associated with worse prescribing quality. These findings may be of use for implementing educational interventions, thereby achieving quality antibiotic prescribing and tackling the bacterial resistance emergency.

Our study indicates that inappropriate antibiotic prescribing in dentistry is a relatively frequent practice. Half of all dentists displayed inappropriate antibiotic prescribing habits in more than 28.6% (10/14) of the clinical situations posed, similar to what has been observed in recent studies [31]. The presence of overprescribing was noteworthy, especially in clinical situations such as (i) irreversible pulpitis with acute apical periodontitis, (ii) pulp necrosis with acute apical periodontitis, (iii) incision and drainage of localised intraoral inflammations, and (iv) necrotising gingivitis, in all cases with an inappropriate prescribing rate of over 50%. These percentages are similar to those identified in other studies, [32] and higher than those described for dentists in the USA [26].

Another group of clinical situations, with incorrect prescribing by a fifth to one third of participants, which are highly relevant from a public health standpoint due to their great frequency in dental practice, are chronic periodontitis and irreversible pulpitis**.** In addition, around 10% of dentists were observed to prescribe antibiotics as a treatment for dental pain, a finding consistent with other papers reporting a prescribing rate of 15% [32, 33]. All this over-prescription seems to be in the context of the overuse of antibiotics in Spain [23, 34–36].

### Related knowledge and attitudes

Our results indicate that fear (OR 1.12, 95% CI:1.07–1.18) of complications seems to worsen prescribing quality, particularly in pulp diseases and prevention of infection. This factor has been indirectly linked by other papers [37–41] to lack of trust when dentists come to prescribe antibiotics and/or have doubts about the diagnosis. Economic benefit also seems to exert a negative influence on prescribing quality in dentistry. Such benefit could be linked to patient loyalty, and satisfaction with the treatment received, as indeed occurs in the case of community pharmacists [23] Similarly, and despite not showing a statistically significant relationship, our results show a trend to inappropriate prescribing, leaning in favour of complacency towards the patient. This is in line with other papers [24]. There are some strategies can help to improve this: deferred prescribing [34] shared decision-making process with regard to patients’ treatment and access to their clinical histories [42].

Perception of the magnitude of the problem of resistance is another factor which this study has identified as influencing the quality of antibiotic prescribing. Health professionals who evinced concern at the advance in resistance obtained better results in prescribing quality, a finding that is in line with those for other healthcare groups, such as physicians [35].

Lastly, lack of time is one of the determinants of antibiotic prescription in previous studies [31, 38, 43], contrary to our study. The high number of dentists and the low demand for dental care observed in our country, could allow dental emergencies to be attended to without interfering with the daily schedule, which could be influencing our results [44].

### Socio-demographic characteristics

Other non-modifiable factors that appear to worsen the quality of antibiotic prescribing are years of experience, practising in the private sector, and postgraduate training in prosthodontics. This finding is in line with those of other studies, which have identified worse knowledge among professionals with a longer intervening period of time since they graduated from university [45]. These results suggest the importance of implementing antimicrobial stewardship programmes dating from the academic preclinical stage, with the aim of creating good prescribing habits from the very earliest point in training [46].

### Implications for practice

This inappropriate prescribing detected in our study appears to indicate the need for dentists to undergo in-depth, improved training in the management of antibiotics and access to clinical guidelines which incorporate available scientific evidence. The lack of consistency between different studies could be accounted for by differences in the indications found in clinical guidelines [27, 32, 33]. Hence, drawing up easily accessible, evidence-based treatment guidelines could be useful tool for enhancing dentists’ knowledge. Such guidelines should be supported by awareness campaigns about the collective benefit of prudent use of antibiotics.

### Limitations

This study has a series of limitations. The sample may not be representative of all dentists in Spain, something that may affect the estimation of the magnitude of the problem, since this could be affected by non-response bias. The responders may be more keenly aware of resistance and therefore engage in better-quality prescribing, something that would amount to an underestimate of inappropriate prescribing. That said, however, we detected a high frequency of inappropriate prescribing, with the result that the real situation may probably be far more worrying. Moreover, when it comes to the determinants of prescribing, we feel that non-participation would not amount to an important limitation because generalisation of results, rather than depending on the representativeness of the sample, depends instead on the mechanism involved in the association with the study phenomenon [47]. In this case, the goal was to associate dentists’ knowledge and attitudes with antibiotic prescribing quality. Hence, despite the fact that there may be a lack of representation of dentists, the attitudes and perceptions associated with prescribing quality do not vary, and we are thus of the opinion that this would not affect the conclusions of the study. The questionnaire used is not full validated because it was not possible to assess the validity criterion, since there is no external gold standard method for measuring attitudes. However, finding statistically significant differences between knowledge/attitudes and prescribing practices, would support the construct validity of this statements, since there is a conceptual framework (model of knowledge, attitudes and practices) that would explain these association.

Lastly, it should also be borne in mind that, in the case of items on knowledge and attitudes, socially acceptable responses may have been obtained, making for social desirability bias [48].

## Conclusions

In the context of the current global emergency surrounding the problem of resistance, dentists play an important role in misuse of antibiotics. I Our study has not only shown the trend towards overprescription of antibiotics in many clinical situations in dentistry, but has also shown that this is linked to potentially modifiable factors. These findings must be borne in mind in designing educational and training programmes for dentists, aimed at improving their antibiotic prescribing. Given that the quantity of antibiotics prescribed by dentists is by no means negligible, this would serve to control antibiotic resistance in future.

## Supplementary Information

**Additional file 1**: **Table S1**. Responses for the dependent variable.

## Data Availability

Data available under request.

## Abbreviations

WHO: World Health Organization
NHS: National health system
STROBE: Strengthening the reporting of observational studies in epidemiology
