# Supplementary material for: Magnitude and determinants of inappropriate prescribing of antibiotics in dentistry: a nation-wide study
Source: Antimicrob Resist Infect Control. 2023 Mar 20;12:20. doi: 10.1186/s13756-023-01225-z (PMC10026418; doi:10.1186/s13756-023-01225-z)
Supplement: Supplementary file 1 — Additional file 1: Table S1. Responses for the dependent variable. [file 13756_2023_1225_MOESM1_ESM.docx]

|  | **Overall** | **"Appropiate prescribing"** | **"Inappropiate prescribing"** |
| --- | --- | --- | --- |
| *Symptomatic irreversible pulpitis (moderate/ severe preoperative symptoms)* | 195 (22.2) | 29 (6.4) | 166 (38.8) |
| *Symptomatic irreversible pulpitis with acute periapical periodontitis (moderate/ severe preoperative symptoms)* | 475 (54.1) | 148 (32.9) | 327 (76.4) |
| *Necrotic pulp with asymptomatic apical periodontitis (no swelling, no/mild preoperative symptoms)* | 143 (16.3) | 15 (3.3) | 128 (29.9) |
| *Necrotic pulp with acute apical periodontitis (no swelling, moderate/severe preoperative symptoms)* | 463 (52.7) | 130 (28.9) | 333 (77.8) |
| *Necrotic pulp with chronic apical periodontitis (sinus trac present, no swelling, no/mild preoperative symptoms)* | 266 (30.3) | 65 (14.4) | 201 (47.0) |
| *Necrotic pulp with acute apical abscess (swelling present, moderate/ severe preoperative symptoms)* | 819 (93.3) | 415 (92.2) | 404 (94.4) |
| *Avulsion* | 521 (59.3) | 276 (61.3) | 245 (57.2) |
| *Postoperative pain after instumentation or obturation* | 100 (11.4) | 19 (4.2) | 81 (18.9) |
| *Incision and drainage localized intraoral swelling* | 442 (50.3) | 165 (36.7) | 277 (64.7) |
| *Postoperative pain* | 73 (8.3) | 7 (1.6) | 66 (15.4) |
| *Prevent infection after dental extraction* | 186 (21.2) | 53 (11.8) | 133 (31.1) |
| *Pericoronitis (no swelling or systemic symptoms)* | 300 (34.2) | 83 (18.4) | 217 (50.7) |
| *Antibiotic prophylaxis to prevent dental implant failure (routine situations)* | 398 (45.3) | 144 (32) | 254 (59.3) |
| *Necrotising ulcerative gingivitis* | 631 (71.9) | 273 (60.7) | 358 (83.6) |

*Table S1 : Responses for the dependent variable*
